# Climate change, species distribution models, and physiological performance metrics: predicting when biogeographic models are likely to fail

**DOI:** 10.1002/ece3.680

**Published:** 2013-08-22

**Authors:** Sarah A Woodin, Thomas J Hilbish, Brian Helmuth, Sierra J Jones, David S Wethey

**Affiliations:** Department of Biological Sciences, University of South CarolinaColumbia, South Carolina

**Keywords:** Biogeography, climate change, niche models, physiological mechanism, physiological performance, temperature

## Abstract

Modeling the biogeographic consequences of climate change requires confidence in model predictions under novel conditions. However, models often fail when extended to new locales, and such instances have been used as evidence of a change in physiological tolerance, that is, a fundamental niche shift. We explore an alternative explanation and propose a method for predicting the likelihood of failure based on physiological performance curves and environmental variance in the original and new environments. We define the transient event margin (TEM) as the gap between energetic performance failure, defined as CT_max_, and the upper lethal limit, defined as LT_max_. If TEM is large relative to environmental fluctuations, models will likely fail in new locales. If TEM is small relative to environmental fluctuations, models are likely to be robust for new locales, even when mechanism is unknown. Using temperature, we predict when biogeographic models are likely to fail and illustrate this with a case study. We suggest that failure is predictable from an understanding of how climate drives nonlethal physiological responses, but for many species such data have not been collected. Successful biogeographic forecasting thus depends on understanding when the mechanisms limiting distribution of a species will differ among geographic regions, or at different times, resulting in realized niche shifts. TEM allows prediction of the likelihood of such model failure.

## Introduction

Species distribution modeling has emerged as a vital tool for predicting, on a spatially explicit basis, the likely impacts of climate change on natural and human managed ecosystems. The goal of biogeographic modeling is to forecast species distributions in new or altered habitats, including invasions into new regions and responses to expected environmental alterations associated with climate change. Predictions of impacts of climate change on the geographic distributions of organisms are being made by two somewhat divergent groups of investigators, biogeographic modelers, and physiological ecologists, using either correlative or mechanistic approaches to predict future distributions (Jeschke and Strayer 2008; Kearney and Porter 2009). One critical assumption of both approaches is that models developed at one location can be applied to novel conditions, either in space or in time; this is the concept of model stationarity in which the mean and variance of the past can be used to predict future conditions and extremes or in terms of biogeography, niche conservatism, or the conservation of the fundamental niche (Wiens and Graham 2005). These concepts explicitly refer to the fundamental or preinteractive niche, and thus focus on requirements and physiological limitations, ignoring constraints due to direct biotic interactions such as predation and competition (Hutchinson 1978). Biogeographic models explore the realized or postinteractive niche (Austin 2007; Peterson and Nakazawa 2008). In a number of cases where biogeographic models based on the historic distribution of species have been used to predict the expected climatic space of a species in new habitats, the model predictions have been invalidated, suggesting (1) a change in the mechanism of limitation in the new environment and thus a change in the realized niche, or (2) a change in the fundamental niche due to an evolutionary shift in the species characteristics (Broennimann et al. 2007; Fitzpatrick et al. 2007 but see Gallagher et al. 2010; Peterson and Nakazawa 2008). The most parsimonious explanation of such failures of biogeographic models would be number one, a change in the realized niche and thus change in limiting factors across biogeographic ranges. Many authors, however, opt for the second interpretation, assuming an evolutionary change and thus alteration of the fundamental niche. This assumes of course that the authors have adequate measures of the fundamental niche in both habitats. If we are to generate robust predictions of how climate change is likely to impact the geographic distributions of species, it is essential to understand when and where biogeographic models are likely to fail, and the reasons underlying these model failures.

Here, we propose a physiology-based approach to estimate *a priori* the likelihood of success or failure of biogeographic model predictions when applied to novel environments. Our approach estimates the probability of change in the mechanism limiting organism distributions using the knowledge of physiological performance curves based on energetics and metrics of environmental variability. This approach thus relies on an exploration of the critical interactions among environmental variation, physiological responses of organisms, and positive energy balance (degree to which total energy available exceeds maintenance energy costs) and indicates the conditions under which biogeographic models are likely to fail.

Predicting the likelihood of failure of biogeographic models when extrapolated to novel environments is critical both for applied and theoretical reasons. First, such failures severely limit the application of biogeographic models in the analysis of future climate scenarios. For example, climate models are used to predict locations where crops will be successful in the future and planning is currently underway to inform the choice of suitable crop species for different locales (Lobell et al. 2008). The quality of those plans depends on understanding the assumptions of the biogeographic models and the likelihood of their predictions across environments. A potentially catastrophic scenario is one where management decisions are based upon incorrect projections of biogeography, resulting in fallacious climate adaptation strategies. Failure of biogeographic models under novel climatic conditions is a common criticism of correlative modeling approaches (Kearney et al. 2008; Peterson and Nakazawa 2008), but as we discuss, mechanistic models may also fail if there is a change in limiting mechanism in the new locale or time period.

Second, the observed failure of biogeographic models is frequently used as *prima facie* evidence that as a result of introduction to a new location, a species has undergone an appreciable alteration in its ecological niche, that is, a fundamental niche shift (Broennimann et al. 2007; Gallagher et al. 2010). Usually such niche shifts are attributed to an evolutionary change in the population as a consequence of founder effects or adaptation to local conditions. Alternatively, some investigators have proposed that multiple genetic races exist within the endemic species distribution and a supposed “niche shift” can be used to diagnose from which of the original races an introduced species has been derived. While physiological and genetic evidence has shown that such founder effects may indeed occur in geographically fragmented populations (Pearson and Raxworthy 2009), and that local adaptation is possible in populations with limited gene flow (Kuo and Sanford 2009), the observed failure of most biogeographic models is more likely to be based on a failure of model assumptions, rather than a change in the fundamental niche. Thus, an “apparent” niche shift may in reality simply reflect a failure to use sufficiently detailed or physiologically relevant environmental metrics (Peterson and Nakazawa 2008; Helmuth et al. 2010), or a failure to consider a change in the limiting mechanism. Either can result in an “apparent” niche shift when in fact the fundamental niche is unchanged and thus niche conservatism has not been violated.

Finally, recent studies have emphasized that the first ecological responses to environmental change may not always comprise changes in simple presence or absence, but instead may be reflected in sublethal changes in growth and reproductive productivity (Hummel et al. 1995; Petes et al. 2007; Beukema et al. 2009). Likewise, changes in ecosystem services can occur well in advance of any major changes in community structure (Mumby et al. 2011). Such impacts may be particularly important to predict for commercially harvested species (Sará et al. 2011; Fly and Hilbish 2013). Tools capable of quantifying the likelihood of such sublethal effects prior to substantial mortality, such as the one we present here, are therefore critical.

A key concept is that the distribution of species can be driven both by short-term exposure to lethal conditions (Jones et al. 2010; Wethey et al. 2011) or by repeated or longer term exposures leading to energetic failures (Jansen et al. 2007; Fly and Hilbish 2013). Here we define the difference between these lethal and sublethal exposure limits as the transient event margin (TEM), the range of environmental conditions below the short-term lethal limit that an organism may endure on a transient basis, but which would lead to mortality over longer time spans. We propose that knowledge of a species' physiological constraints in terms of performance and lethal limits, that is, TEM, coupled with measures of the degree of environmental variation in relevant limiting factors allows one to predict whether a biogeographic model is likely to fail when applied to a new location or time. In the following sections, we describe the relationship of TEM to physiological performance curves, how environmental variance interacts with TEM to allow *a priori* predictions of biogeographic model failure, and illustrate this concept with both a case study and predicted patterns for combinations of TEM and environmental variance. We apply physiological theory directly to the question of how we model biogeographic responses of populations to environmental change, and what consequences this has for the likelihood of biogeographic model success.

## TEM Concept, Performance Curves, and Climate Change

For the purposes of our discussion, we confine our comments to those models directly linked to energetics because in addition to mortality, it is the ability of the individual to grow and reproduce that determines large-scale biogeographic responses to environmental change (MacArthur 1972). Similarly, we confine ourselves to responses to temperature given its link to climate, and its importance as a fundamental driver of physiological rates and performance (Somero 2002; Dell et al. 2011; Huey and Kingsolver 2011). However, our conclusions are broadly applicable to other environmental drivers of physiological performance.

### Performance curves and range limits

**Figure 1:**
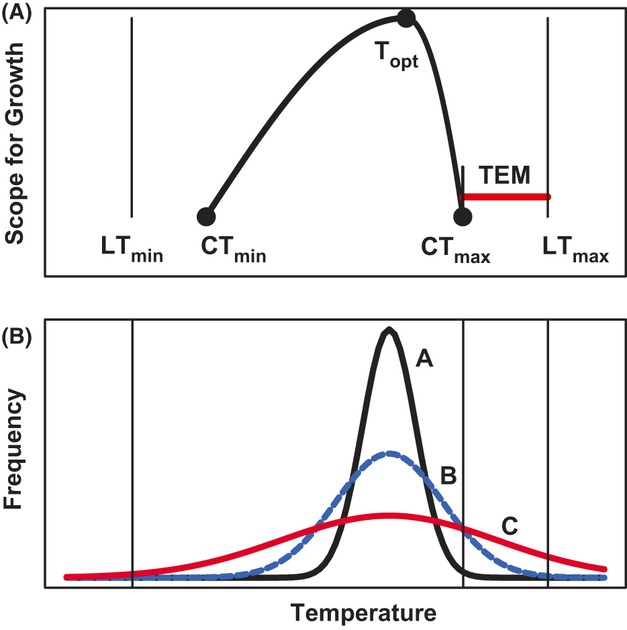
(A) Relation between performance as scope for growth (SFG) and temperature. Lethal limits are LT_min_ and LT_max_. Performance limits are the critical minimum temperature (CT_min_) and critical maximum temperature (CT_max_), the body temperature at any point in space and time is T_body_ and the optimal temperature is T_opt_. The upper transient event margin (TEM) = LT_max_ − CT_max_. (B) Temperature versus frequency for environments A, B, and C. TEM from part A expressed on yearly environmental variance curves for three environments, all with the same mean environmental temperature, but differing in variance.

The relationship between physiological (and ecological) performance and body temperature of most species is displayed as a thermal performance curve (Fig. 1A) (Martin and Huey 2008; Angilletta et al. 2010). When organism body temperatures are below their optimum for performance (T_opt_), performance increases gently with increasing temperature, but after reaching a maximal value (T_opt_), performance declines, often sharply, to a zero value (CT_max_) in response to even small increases in temperature (i.e., once temperatures extend beyond the species “pejus” temperature, Pörtner 2010). These curves are often “left-skewed” and have been reported for an array of species (lizards, clams, trees, snails, etc.) and for the performance of many different physiological functions (sprint speeds, heart beat rate, pumping rate, photosynthesis, scope for growth, etc.) and thus appear to be a general response to temperature (Angilletta et al. 2010).

With regard to the responses of species and communities to climate change, the consequences of strongly left-skewed performance curves are profound. First, as discussed by Deutsch et al. (2008), Martin and Huey (2008) and others in terms of adaptive thermoregulation theory, the shape of these functions predicts that small increases in body temperature may cause a highly successful and productive population of organisms functioning near T_opt_ to become unsuccessful abruptly. Thus, small increases in environmental temperature in the future, that is, gradual warming may lead to large and abrupt changes in distribution and abundance, even when no impacts in terms of population dynamics had previously been observed. Dillon et al. (2010), Beukema et al. (2009), and Southward et al. (1995) all present data illustrative of this pattern. Second, the point at which physiological performance falls to zero (CT_max_) is not equivalent to the upper lethal limit (LT_max_). Range limits can thus be set either by short duration, lethal exposures (body temperature ≥ LT_max_:Wethey 2002; Jones et al. 2010) or by more chronic exposures to temperatures above CT_max_, but below LT_max_ that lead to degradation in vital activities such as reproduction with impacts for population dynamics or food-gathering abilities with implications for energy balance (CT_max_ ≤ body temperature < LT_max_: Fig. 1A). Importantly, the shape (width or steepness) of the performance curve and the values of T_opt_ and CT_max_ vary among species and are measures of thermal tolerance (Somero 2002).

### Scope for growth versus temperature curves

For a physiological performance curve of scope for growth (SFG: energy in excess of maintenance energy [Warren and Davis 1967]) versus temperature, TEM is the environmental space bounded on one side by the upper critical limit for the species expressed in terms of energetics (Fig. 1A: CT_max_), and on the other by the lethal limit (Fig. 1A: LT_max_). There is also a comparable TEM between CT_min_ and LT_min_, but we will confine ourselves to TEM at high temperatures here. For such curves, CT_max_ is the point at which the organism enters negative energy balance, the end of positive secondary production for consumers, or net primary productivity for primary producers; LT_max_ is the point at which the organisms die even after short-term exposure (Fig. 1A). TEM is the environmental space within which individuals can exist, but not indefinitely, given that they are in negative energy balance. A number of organisms have negative energy balance under thermal conditions that do not exceed their short-term lethal limit (e.g., clams: *Macoma balthica*: Jansen et al. 2007; mussels: *Mytilus edulis*: Widdows and Bayne 1971; *Mytilus galloprovincialis*: Sará et al. 2011; *Mytilus trossulus*: Fly and Hilbish 2013; worms: Sommer and Pörtner 1999; plants: Loveys et al. 2002; insects: Kingsolver et al. 2006), which lead to reductions in tissue weight, failure of reproduction, and in some cases death.

### TEM and environmental variation

The likelihood of an individual finding itself within TEM space is clearly a function of both mean body temperature (average position on thermal performance curve) of individuals at that location as well as the scale of environmental fluctuations (variability from the mean) for that location relative to the size of the TEM as illustrated by Figure 1. Consider a single species comprising multiple populations living under three different climatic conditions. Individuals of the species living with the smallest environmental variance (Fig. 1B: environment A), and thus the lowest variability in body temperatures, rarely are exposed to conditions of negative SFG much less than lethal temperatures. Those living in the more variable environment B often enter TEM space due to the greater environmental variance (Fig. 1B). Those living in the most variable environment (“C”, described by the more platykurtic curve), pass through TEM space and often exceed its boundary at LT_max_ due to the level of environmental variance and thus die (Fig. 1B: environment C: 10% frequency of temperature exceeding LT_max_).

According to adaptive thermoregulation theory, thermal generalists have broad performance curves, shallower slopes, and lower performance peaks, whereas thermal specialists are expected to have narrower performance curves, steeper slopes, and higher performance peaks (Huey and Slatkin 1976; Martin and Huey 2008). Thermal generalists are expected to be common in highly variable thermal environments, and thermal specialists in less variable thermal environments (Fig. 1B: more variable: environment C vs. less variable: environment A). Similarly, for species characteristic of highly variable environments, we expect TEM to be large relative to environmental fluctuations and we expect the decay slope, or right side of the curve, to be relatively shallow, reflecting the ability of the organism to survive the rate of energy consumption during the transient period of negative energy (Table 1). Environments of this type include those that are essentially biphasic, such as terrestrial habitats with large diurnal environmental regimes including high-altitude locales even in the tropics (Ghalambor et al. 2006) and intertidal habitats with large tidal excursions during times when aerial and submerged body temperatures are not congruent (Mislan et al. 2009; Pincebourde et al. 2012). For organisms common to less variable environments, we expect the slope on the right side of the productivity curve to be steep in the absence of selection to maintain performance in the face of environmental variation (Stillman and Somero 2000) (Table 1).

**Table 1:** Expected patterns of physiological performance curves (PPerform) in environments differing in degree of environmental variation

	Relative environmental variation	
	
	Small	Large
TEM size	?	TEM > scale of environmental variation
Decay slope	Steep	Shallow

Predictions are for size of TEM and steepness of slope of the right side of the physiological performance curve (“decay slope”). “?” no clear prediction for size of TEM relative to scale of environmental variation.

If TEM is small (∼0) in absolute terms or small relative to environmental variation in a region with high environmental variability, increases in temperature beyond T_opt_ are expected to cause rapid collapse in the population as nearly simultaneously performance limits (CT_max_) and lethal limits (LT_max_) are exceeded. We predict that such species will be especially amenable to ecological forecasting using correlative or niche envelope approaches involving minimal physiological information because there is a narrow range of temperature over which all tolerances are exceeded (Table 2); mechanistic models will also be successful as the failure point is similar for all mechanisms. All these models should yield similar predictions for points of collapse (Table 2). In contrast, if TEM is large relative to environmental fluctuations, then negative energy balance is likely to occur without direct lethal limits being exceeded, except during extreme (and thus rare) events. In this case, predictions based only on lethal limits (many correlative niche models) and those based on performance are expected to yield strikingly different predictions (Kearney et al. 2008). Similarly, mechanistic models may also fail if the mechanism of limitation changes with the environment such as a move from environment “C” (lethal limits are often exceeded) to “B” (lethal limits are more rarely exceeded, but being in negative energy balance is common) would likely entail (Fig. 1B). In such a scenario, only models that include consideration of both LT_max_ and CT_max_ will be successful.

**Table 2:** Expected success of biogeographic models based on similarity of the environment for which the model was developed and the new environment for which predictions are being made

Environment comparison	Model success
Environ variance >> LT_max_, both environments	Success
TEM = 0, both environments	Success
TEM >> 0 in one but not both environments	Failure likely^1^
TEM >> 0 in both environments, but environ variance < LT_max_ in at least one environment	Failure likely^1^

“Environ variance” is environmental variance.

1 Failure can occur in at least three divergent ways due to differences between the two environments: negative SFG, lethal temperature, change in mechanism. See text.

The TEM explicitly distinguishes between conditions leading to chronically poor ecological performance and directly lethal conditions. This allows exploration of several important consequences of environmental change, including likelihood of reproductive failure and thus reduced or nonexistent reproductive value given changes in climate. Given a large TEM and a shallow slope of the performance decay curve, organisms may be able to endure a low ecological performance or negative energy balance for a substantial period of time without mortality; however, one would expect loss of tissue and potential failure of reproduction (see data in Petes et al. 2007; Honkoop and Beukema 1997; Lucas and Crisp 1987). An interesting consequence of large values of TEM relative to the scale of environmental variation, therefore, is that reproductive failure will be commonly observed near the species limits of performance (Table 3). Species with large values of TEM persist under conditions of very low or negative performance when the adults are likely to consume the reproductive reserves. In contrast, for species with small TEM values relative to normal environmental fluctuations, reproductive failure will rarely be observed, rather lethality will follow closely any collapse of physiological performance (Tables 3). Species for which demographers invoke storage effects, the presence of old individuals living near the range limits of the species which rarely reproduce, but extend the reported range of the species (e.g., *Semibalanus balanoides*: Wethey and Woodin 2008), are again likely to have large TEM values. Therefore, the contrasting limiting biogeographic mechanisms of adult survival versus juvenile recruitment proposed by Hutchins (1947) may derive from differences in TEM and environmental variability. In sum, if storage effects or reproductive failure is commonly observed for a species near its range limits, the TEM is likely to be large relative to environmental fluctuations and knowledge of mechanism in each geographic locale will be critical to the success of extending predictions in space and time (Table 2).

**Table 3:** Likelihood of observation of reproductive failure or storage effects in populations given the size of TEM and scale of environmental variation

	TEM ∼0	TEM >> 0
Environ variance > TEM	Very low	Unlikely
Environ variance < TEM	NA	High

## Case Study

### Environment: Atlantic coastlines, continental versus oceanic climates

**Figure 2:**
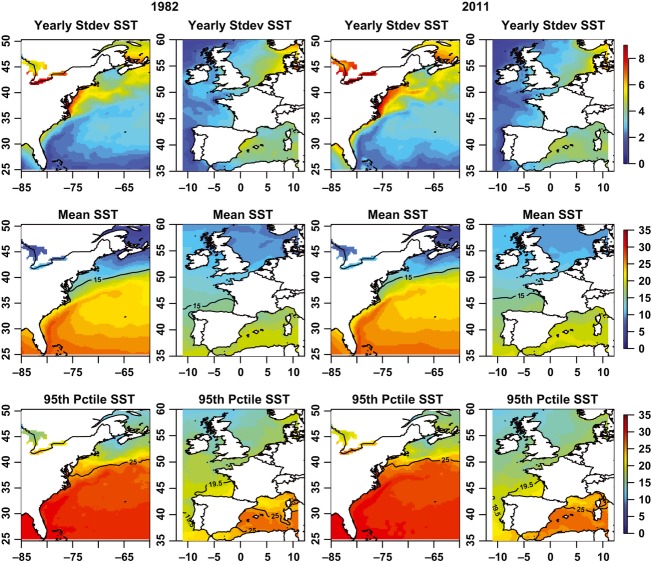
SST values from Daily Optimally Interpolated SST (Reynolds et al. 2007) for the Atlantic coasts of North America and Europe for 1982 and 2011. For each location and year in order are yearly standard deviation in SST values, mean SST value, and 95th percentile SST value. Scale bars in degrees centigrade; cool colors indicate lower value.

A physical example in both space and time of environments differing in variance is given in Figure 2 which illustrates the dramatically different thermal conditions of the Atlantic coasts of North America and Europe, environments that share a number of common species including barnacles, mussels, and algae. The western Atlantic margin (North American coast) has a continental climate with high within-year variation in sea surface temperature (SST), and the eastern Atlantic margin (European coast) has an oceanic climate with low within-year SST variation (van den Hoek 1982; Jenkins et al. 2008). This can be seen most easily in the maps of annual standard deviations of SST on the two sides of the Atlantic. Most of the coast of Europe has an annual standard deviation of 2 to 4°C, whereas the North American Atlantic coast has a standard deviation of 4 to 8°C (Fig. 2, row 1). This translates into a confluence of extreme highs and lows in the western Atlantic, and a confluence of year-round moderate temperatures in the eastern Atlantic. On the Atlantic coast of North America, an annual mean SST of 15°C was recorded at sites near 36.5°N in both 1982 and 2011 (Fig. 2, row 2). The 95th percentile of daily temperatures (Fig. 2, row 3) is a measure of the hottest conditions experienced during each year. For the same pixels with an annual mean temperature of 15°C, the 95th percentile was ∼25°C, indicating that on the North American Atlantic coast some of the hottest SST values were 10°C hotter than the annual mean at the same location (Fig. 2, rows 2 and 3). On the Atlantic coast of Europe, conditions were very different, with much less variation in annual temperature. An annual mean temperature of 15°C occurred at approximately 45°N in 1982 and 2011 on the European coast, but the 25°C contour on the 95th percentile map was in the Mediterranean, over 1000 km away (Fig. 2, rows 2 and 3). The actual 95th percentile value for the pixels at 45°N on the European coast with an annual mean of 15°C was 19.5°C, 5.5 degrees less than that on the North American coast (Fig. 2). Put another way, the spatial gradient in temperatures was much steeper on the western Atlantic than in the eastern Atlantic (Lima and Wethey 2012). Temporal variation between 1982 and 2011 was also very different for the two coasts. On the coast of North America, the yearly standard deviation of SST (Fig. 2: row 1) was much higher in 2011 than in 1982, particularly in the mid-Atlantic region. In contrast, the yearly standard deviation of SST for the Atlantic coast of Europe was approximately the same in 1982 as in 2011 (Fig. 2: row 1). For many organisms, the contrast between the coasts represents environments of type “A” (eastern Atlantic) and type “C” (western Atlantic) of Figure 1B; therefore, one would expect a change in mechanism of limitation of distribution when moving between the two coastlines as reported for several species of marine algae (van den Hoek 1982). One would also expect smaller biogeographic ranges on the western ocean basin given the much more rapid spatial change in isotherms (Fig. 2); such a prediction is consistent with the findings of Jenkins et al. (2008), Schmidt et al. (2008), and van den Hoek (1982).

### Organism: *Mytilus edulis*

**Figure 3:**
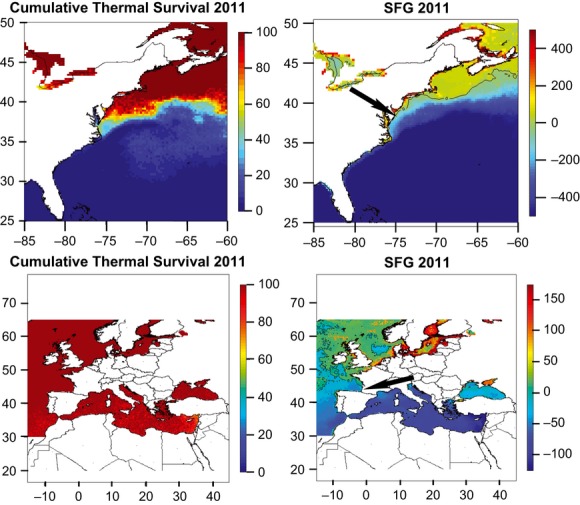
Geographic limits of *Mytilus edulis* predicted from daily adult mortality due to high water temperatures (left) or energetics (right) (SFG = scope for growth) in 2011. Black arrows indicate actual geographic limits in North America (top right) and Europe (bottom right), see text. Scale on left: percent survival based on temperature, scale on right: SFG in calories/gram dry weight/year (Bayne et al. 1976). Cool colors indicate lower success. SFG contour lines indicate annual zero SFG. Maps generated from Reynolds optimally interpolated 2011 daily SST (Reynolds et al. 2007, http://nomads.ncdc.noaa.gov) and 2011 monthly average MODIS Aqua chlorophyll concentrations (http://oceancolor.gsfc.nasa.gov) in conjunction with relationships between survival rate and water temperature (Jones et al. 2010) or among water temperature, food ration, and scope for growth (Bayne et al. 1976).

Few datasets exist that allow testing of the predictions of Table 2. This is not surprising given the necessity of knowing both CT_max_ for SFG and LT_max_ to define TEM plus the degree of environmental variation in both the environment in which a biogeographic model is originally developed and the new environment to which it will be applied. However, the work of Jones (2010) on geographic determinants of the ecologically dominant marine mussel *Mytilus edulis* is an excellent example. Jones (2010) presented a mechanistic biogeographic model for *M. edulis* on the Atlantic coast of North America based on LT_max_ which was calculated experimentally in both air and water and measured in the field (Jones et al. 2009, 2010). Physiological limits were then compared against environmental temperatures, and the biogeographic distribution of this species on the east coast of North America was accurately predicted (Jones 2010) (Fig. 3). The hindcast done with the model also successfully predicted historical changes in distribution over the past 50 years (Jones et al. 2009, 2010). When this same model was applied in Europe, using physiological data for the environment of origin, it failed utterly to predict the biogeography of this species; the observed distribution of the species in Europe was ∼50% less than that predicted by the model (Fig. 3) (Jones 2010). The model from the eastern Atlantic based on thermal tolerance limits, LT_max_, predicted a distribution including the Mediterranean and the Black Sea; the actual geographic limit of *M. edulis* on the Atlantic coast of Europe is near Arcachon, France (Hilbish et al. 2012). However, when an energetics model (Fig. 3, bottom right panel) derived from Bayne et al. (1976) is applied to Europe, the range limit predicted is close to the observed limit at Arcachon (Fig. 3: arrow lower right panel). Thus, on the East Coast of North America, lethal limits appear sufficient to predict accurately both historic and contemporary species range boundaries. In stark contrast, in Europe, observed biogeography cannot be accurately predicted without consideration of physiological performance based on energetics (i.e., balance between income from food supply with expenditures due to respiration). These results are consistent with those of Sará et al. (2011) who showed that Mediterranean *M. galloprovincialis* intertidal distributions are set by energetic failure at some locations, but by lethal exposures at others and with those of van den Hoek (1982) for a number of species of marine algae inhabiting shores of both the eastern and the western Atlantic. For *M. edulis*, there is an appreciable TEM (7–10°C) between the temperature at which energetic performance declines (*M. edulis* cannot maintain a positive energy balance above 20–23°C water temperature depending upon food availability: Fly and Hilbish 2013; Bayne et al. 1976) and its thermal tolerance limit (∼30°C: Sorte et al. 2011; Jones et al. 2009, 2010). Its failure during warm winters (Honkoop and Beukema 1997) in terms of relative weight loss parallels its performance failure in warm summers. The implication is that in the western Atlantic, the warming rate of the continental climate is so rapid that CT_max_ and LT_max_ are both exceeded over a short enough time that the animals experience lethal conditions before they run out of energy, that is, TEM >> 0, but environmental variance >> TEM (Table 2). In the oceanic climate of the eastern Atlantic with much less environmental variance (Fig. 2), LT_max_ is rarely, if ever, exceeded, but individuals living from northern Spain through the Mediterranean suffer conditions above CT_max_ and die from negative energy balance, that is, TEM >> 0 and TEM >> environmental variance (Table 2). In both cases, temperature limits are important, but the prediction based on LT_max_ alone is inadequate to predict the geographic range of *M. edulis* in the eastern Atlantic congruent with our predictions based on the scale of TEM relative to environmental variation. Given currently available environmental data and some knowledge of parameters likely to limit organism distributions, one can address the question of the scale of environmental variation in the two environments. In this case, one is an oceanic climate and one is a continental climate; so, the scale of environmental variation is not even remotely similar (Fig. 2). Table 2 suggests immediately the need for caution in application of models across such a scale difference.

## General Predictions

The concept outlined in this study has both general and specific value. The general hypothesis is that the magnitude of the disparity (TEM) between performance and tolerance temperature thresholds relative to environmental variance determines the likelihood of failure of biogeographic model predictions. Specifically, whereas correlative (climate envelope) models may be sufficient for species with small TEM or habitats with much larger environmental variance than the scale of TEM, more process-based models – and especially those that incorporate energetics approaches (e.g., Sará et al. 2011) – may be required for species with large TEM values relative to environmental variance. Importantly, data on performance, energetics, and environmental variation allow exploration of the likelihood of changes in mechanism; mechanistic models can also fail if the possibility of change in limiting mechanism is not explored (Table 2).

This analysis yields a number of explicit predictions under warming conditions:

- if environmental variance increases for any reason, this will reduce the scale of TEM relative to environmental variance and rapid changes in abundance well within current species range boundaries may occur as CT_max_ is exceeded repeatedly.
- if environmental variance is reduced for any reason, this will increase the scale of TEM relative to environmental variance and energy-based performance metrics will be much more important to the likelihood of biogeographic model success.
- biogeographic models for a species are most likely to fail when applied to regions outside the area for which the model was developed, particularly if TEM is large and the scale of environmental variance differs across environments as illustrated by Figure 2.
- regardless of mechanism, biogeographic model success is expected to be higher in species with small values of TEM, that is, similarity of lethal tolerance and performance thermal limits.
- relative magnitudes of TEM for interacting species will drive dramatic releases from competition and predation, yielding performance changes unrelated to direct temperature effects, but rather to the relative scales of their TEMs and the environmental variance. Specifically, a reduction in the capacity of a predator or competitor will enhance the capacity of the corresponding species, that is, increase its realized niche. This can be seen in the contrast between north- and south-facing rocks in intertidal zones where the inferior competitor for space expands its distribution as the physiological limits of the superior competitor are reached sooner due to greater insolation (Wethey 2002). Similarly, feeding rates are reduced in predators exposed to temperatures > T_opt_, but <LT_max_ (Pincebourde et al. 2008) with potential positive effects on the prey.
- reproductive failure and storage effects will be much more common in species with large values of TEM than in those where performance and lethality are congruent. As discussed earlier, as a species exists within TEM space with a negative energy budget, the likelihood of reproduction is low (Jansen et al. 2007; Petes et al. 2007).

## Measures of TEM

Tests of these hypotheses will require better data on such parameters as CT_max_ and lethal limits so that TEM can be determined. Measurement is not a trivial problem. In terms of energy, CT_max_ is the point at which energy balance becomes negative. A variety of surrogates have been used to determine this such as the onset of muscle spasms, common in the vertebrate literature following the lead of Lutterschmidt and Hutchison (1997), or in gastropods inability to crawl or onset of heat coma, which is defined as the loss of ability to hold onto a surface and loss of response to prodding (Table 4). If food is available and failure of positive energy balance reflects the lack of the ability of the organism to capture prey at these temperatures – as is true of all these surrogates, then the surrogates should suffice. However, if food is not available and the organism continues to expend energy in activity due to thermal conditions, resulting in negative energy balance, then surrogates such as muscle spasms or heat coma are inappropriate; negative energy balance will occur at lower temperatures than those that cause loss of motor function. There are numerous examples of organisms losing tissue mass by maintaining activity in the absence of food (e.g., Honkoop and van der Meer 1997; Beukema et al. 2009). The surrogates will suffice as indicators of CT_max_, but if and only if food is available. If food is not available, then actual measurements of energy balance such as scope for growth or dynamic energy budget approaches will be necessary (e.g., Sará et al. 2011; Fly and Hilbish 2013).

**Table 4:** Thermal performance and lethal limits for three species of the marine gastropod *Nucella*

Species	Thermal limits measured in degrees C			

	Crawling	Heat coma	Lethal limit	TEM
*N. ostrina*	>30^1^	31^1^ or 32 to 36^2^	34 to 35^1^	<5 crawling 0 to 3 heat coma
*N. canaliculata*	No data	22 to 25^2^	32^3^ to 33^2^	8 to 11 heat coma
*N. lamellosa*	30^1^ small 25^1^ large	27^1^	32^2^ to 33^1^	2.5 crawling—small 7.5 crawling—large 5.5 heat coma
*N. lapillus*	No data	30^4^	26 to 31^5^, 35^6^ 36^4^	0 to 6 heat coma^4^

Results are laboratory measurements with the exception of Bertness and Schneider (1976). Laboratory snail sizes correspond to “small” of Bertness and Schneider (1976). Range of values often reflect data on animals from different tidal heights and exposure times or seasons (Davenport and Davenport 2005, 2007; Sorte and Hofmann 2005). Growth rates are also reduced at higher temperatures (e.g., *N. ostrina* reduced growth ≥28°C: Dahlhoff et al. 2001).

1 Bertness and Schneider 1976

2 Sorte and Hofmann 2005

3 Kuo and Sanford 2009

4 Sandison 1967

5 Davenport and Davenport 2005, 2007

6 Gibson 1970

**Figure 4:**
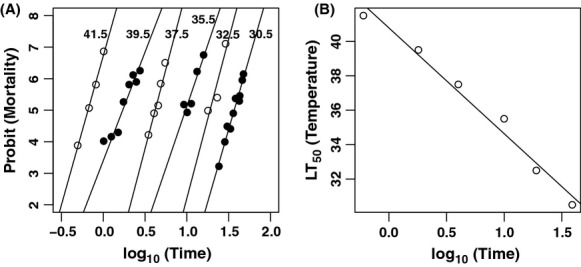
Relationship between mortality rate, temperature, and exposure time in the barnacle *Semibalanus balanoides*. Data replotted from Foster (1969). (A) probit of mortality plotted as a function of the log_10_ of exposure time. Each line represents a different exposure temperature. Mortality is 95% at a probit of 7, 50% at a probit of 5, and 5% at a probit of three. Exposure temperatures are listed adjacent to the lines. (B) LT_50_, the temperature at which 50% of the population dies, as a function of the log_10_ of exposure time. These data were determined from A as the points on each of the lines where probit (mortality) = 5.

An additional difficulty with the estimation of CT_max_ and therefore of TEM is that there is no standard method for estimating lethal temperature conditions (Santos et al. 2011). For many animals (Foster 1969), there is a log-normal distribution of survival times at any particular temperature (Fig. 4A), as well as a log-linear relationship between exposure time and the temperature at which 50% of the population dies (Fig. 4B). In the case of Foster's (1969) data on the barnacle *Semibalanus balanoides*, 50% of the population dies in 0.6 h at 41.5°C, in 10 h at 35.5°C, and in 39 h at 30.5°C (Fig. 4B), so one could claim any LT_50_ temperature between 35.5 and 41.5 as LT_max_ as ecologically relevant, depending upon whether one was considering exposure to the high temperature of the day, or to sustained high temperatures during a 10-h low tide on a hot day. At the upper limit of *S. balanoides* in the intertidal on Horse Island, Connecticut (1.9 m above mean lower low water (MLLW), 41° 14.62 N, 72° 45.54 W), for example, *S. balanoides* may experience >24 h aerial exposure during calm neap tides, whereas at the center of the intertidal distribution (1.4 m above MLLW) they may only experience ∼6 h exposures during the same tides (Wethey 2002). Most authors appear to use the center of the distribution as the exposure point, but not all do so. Additionally, collection locale both within and across sites will affect the results as is evident in Table 4 for the gastropod *Nucella*. Note the range of reported values for lethal limits and heat coma temperature; these correspond to both micro- and macrosite differences which affect both acclimation and thermal history (Pörtner 2012). Thermal history is seen in the data for *Nucella* in Table 4, where prior exposures to elevated, but not lethal temperatures, can in turn raise LT_max_ and thus may “prepare” organisms for even more extreme exposures (Dong et al. 2008, but see Jones et al. 2009 for the reverse result). Variability within populations to thermal sensitivity, both due to different thermal histories and genetic variance within any given population, may be crucial to understand (Schmidt and Rand 2001) if we are to predict impacts of climate change. Depending on reproductive mode and thus likely dispersal distance, there may be a genetic basis to variability in thermal sensitivity that can be geographically complex as also seen in *Nucella* (Kirby et al. 1994; Kuo and Sanford 2009). The variance in TEM in Table 4 for species of *Nucella* reflects both changes latitudinally and within habitat as a function of exposure and season as well as the metrics used and reveals the necessity to standardize or at least specify clearly how data are collected.

## Conclusions

Two critical hypotheses form the foundation of most biogeographic modeling: (1) the assumption of stationarity or niche conservatism in space and time (predictions made on the basis of one location and time will be valid in other geographic regions at other times; conservation of mechanism and no change in the fundamental niche), and (2) the assumption that the same mechanisms that limit the distribution of a species in one geographic locale or epoch are the limiting mechanisms in all other parts of the species' geographic range or all other epochs. These hypotheses are the underlying assumptions of all correlative niche-based and most mechanism-based models of geographic distribution of species. If they are falsified, we will need fundamentally new approaches to forecasting biogeographic responses to climate change. Our initial results and analysis call into question the assumptions of stationarity or conservation of limiting mechanism, but only if TEM is large relative to the scale of environmental variation (Tables 3). When TEM is large, our analysis indicates that it is essential to understand both the mechanisms responsible for biogeographic distribution and the geographic distribution of those mechanisms (see Fig. 3). Understanding such mechanisms may be particularly critical and daunting because climate change will likely expose populations to novel conditions, and because variability in environmental drivers is expected to increase. However, if our analysis is correct, then the size of TEM allows one to estimate the likelihood of such failure. A number of indicators appear to be correlated with large TEM values such as observation of reproductive failure or demographic storage effects near range boundaries and relatively shallow decay slopes toward CT_max_. Again, those metrics yield predictions of the likelihood of failure of biogeographic model extensions in time or space (Tables 3). A key to successful biogeographic forecasting is thus an understanding of when detailed mechanistic knowledge is essential and when the mechanism is likely to change. The relationship between the TEM and the environmental variance provides an important indicator of success.
